# Esophageal Perforation With Right-Sided Hemothorax in a Patient With Suspected Variceal Bleeding: A Diagnostic Challenge

**DOI:** 10.7759/cureus.105270

**Published:** 2026-03-15

**Authors:** Navid Moghimi, Jan Krzak, Per Helligsø, Biniam B Teklay

**Affiliations:** 1 General and Colorectal Surgery, University Hospital of Southern Denmark, Aabenraa, DNK

**Keywords:** boerhaave syndrome, esophageal perforation, gastro-esophageal surgery, hemothorax, upper gastrointestinal bleeding

## Abstract

Esophageal perforation (Boerhaave syndrome) is a rare but life-threatening condition that may mimic other causes of upper gastrointestinal bleeding, making early diagnosis challenging. We report a 76-year-old woman with disseminated breast cancer who presented with massive hematemesis and profound hemodynamic instability, initially suspected to represent variceal bleeding. Emergency endoscopy achieved hemostasis at the gastroesophageal junction but did not identify a perforation. During anesthesia, point-of-care ultrasound revealed a large right-sided pleural effusion, prompting CT imaging. CT demonstrated a massive right-sided hydropneumothorax, complete lung collapse, and mediastinal air consistent with esophageal perforation. Despite stabilization and broad-spectrum antibiotics, the patient developed sepsis. Given her advanced malignancy, frailty, and poor prognosis, invasive interventions were withheld and care was transitioned to a palliative approach. This case highlights the diagnostic difficulty of distinguishing Boerhaave syndrome from other causes of massive hematemesis and underscores the value of CT imaging when endoscopy is inconclusive.

## Introduction

Spontaneous esophageal perforation, or Boerhaave syndrome, is a rare but life-threatening condition with an estimated incidence of approximately 3.1 per 1,000,000 per year [1]. It typically results from a sudden increase in intraesophageal pressure following forceful vomiting, leading to transmural rupture of the esophageal wall [2]. The clinical presentation is often nonspecific and may mimic other acute thoracic or abdominal conditions, including myocardial infarction, perforated peptic ulcer, pulmonary embolism, or upper gastrointestinal bleeding [3]. Mortality remains high, particularly when diagnosis is delayed beyond 24 hours [4].

The classical Mackler’s triad, vomiting, chest pain, and subcutaneous emphysema, is described in esophageal perforation but is present in fewer than half of cases, contributing to diagnostic difficulty [5]. Although endoscopy plays a central role in the evaluation of upper gastrointestinal bleeding, its diagnostic usefulness in suspected esophageal perforation is debated due to the risk of exacerbating an existing rupture [6]. In contrast, computed tomography (CT) has become the preferred imaging modality for detecting mediastinal air, pleural effusion, and associated thoracic complications [7].

Given the massive hematemesis and profound hemodynamic instability, variceal bleeding was initially suspected, as this is a common consideration in patients presenting with severe upper gastrointestinal bleeding. We report a case of esophageal perforation presenting as massive hematemesis in a multimorbid patient with disseminated breast cancer, illustrating important diagnostic pitfalls and the decisive role of CT imaging when endoscopy is inconclusive.

## Case presentation

A 76-year-old woman with disseminated breast cancer receiving palliative care was found at home by ambulance personnel after having vomited what was described as a substantial amount of blood. Initial blood pressure was approximately 45 mmHg systolic, rising only to 50-60 mmHg after fluid resuscitation. On arrival to the emergency department, she was hemodynamically unstable, with hemoglobin of 6.2 g/dL and elevated lactate. The patient also reported chest pain on admission. She was treated with intravenous fluids, terlipressin, a proton pump inhibitor, and a transfusion of packed red blood cells. A nasogastric tube drained approximately 1 L of blood-stained fluid.

**Figure 1:**
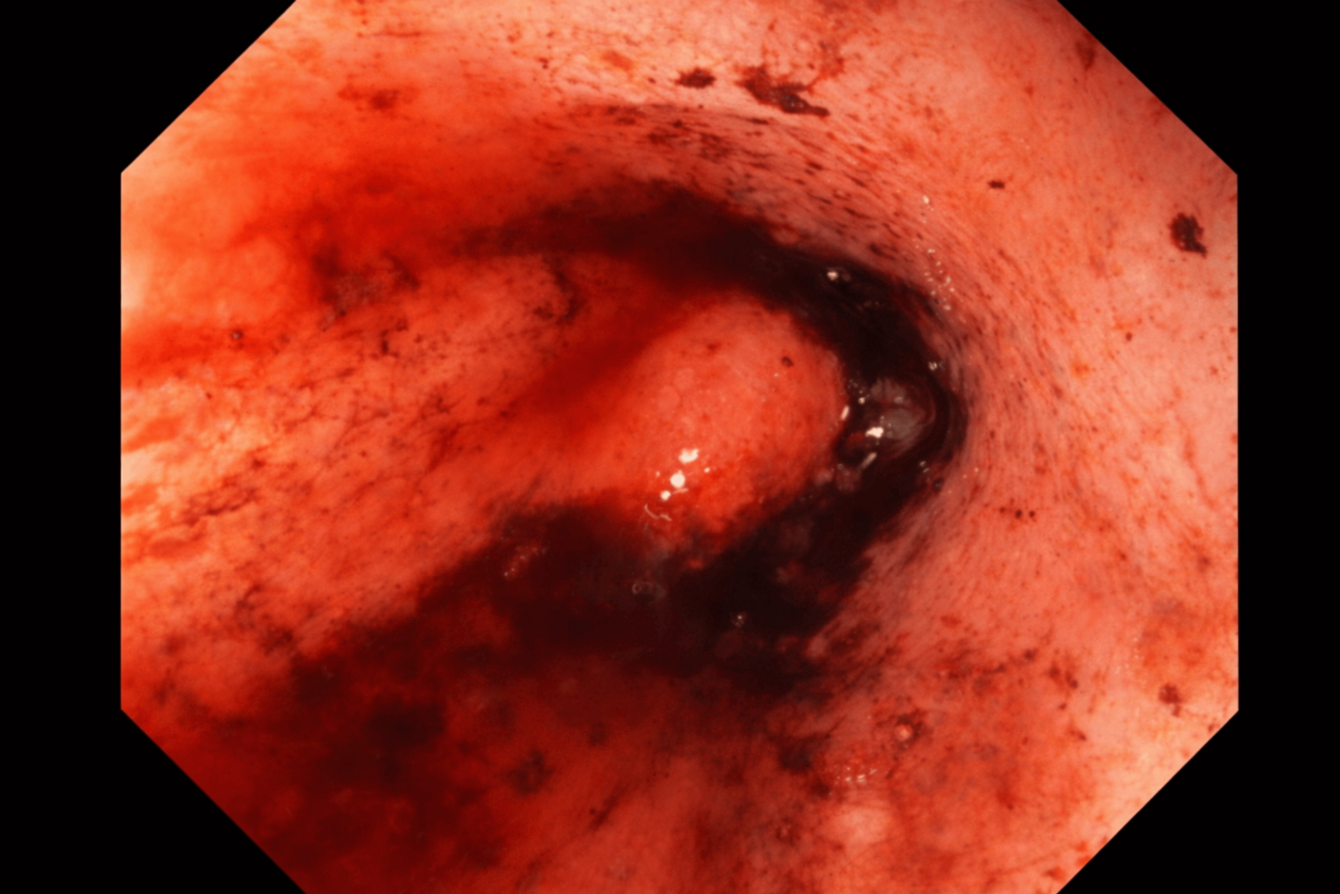
Endoscopic images showing active bleeding at the gastroesophageal junction adjacent to a polyp.

Urgent upper endoscopy demonstrated active bleeding at the gastroesophageal junction adjacent to a large polyp. Hemostasis was achieved using adrenaline injection, diathermy, and multiple clips (Figure 1). The polyp was not considered the primary source of bleeding and was not further evaluated given the acute clinical situation. No clear perforation was identified endoscopically.

**Figure 2:**
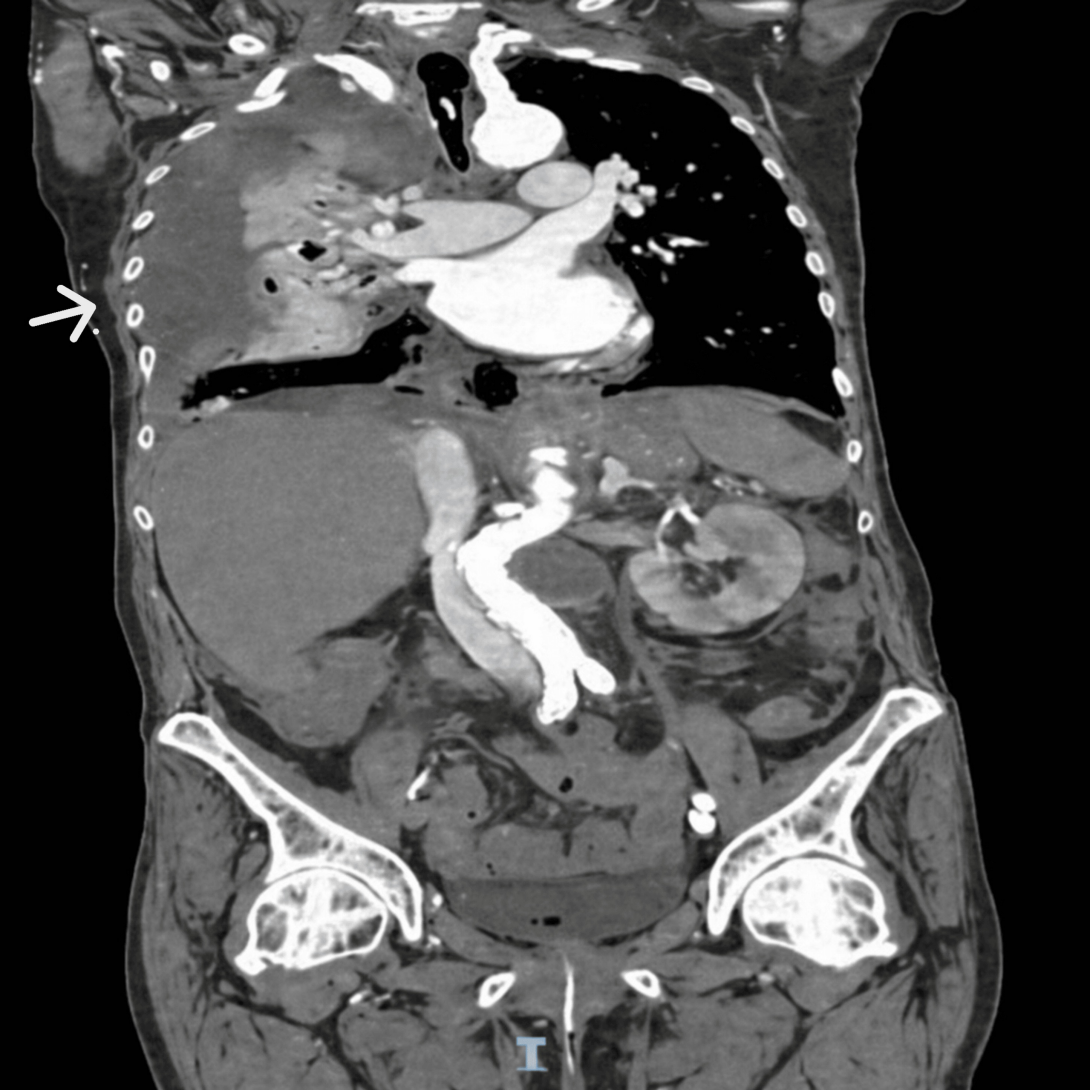
Coronal CT (venous phase) reconstruction demonstrates the extent of right-sided hydropneumothorax and minimal left pleural effusion. The posterior mediastinal air is again visualized (arrow), supporting the suspicion of esophageal rupture.

**Figure 3:**
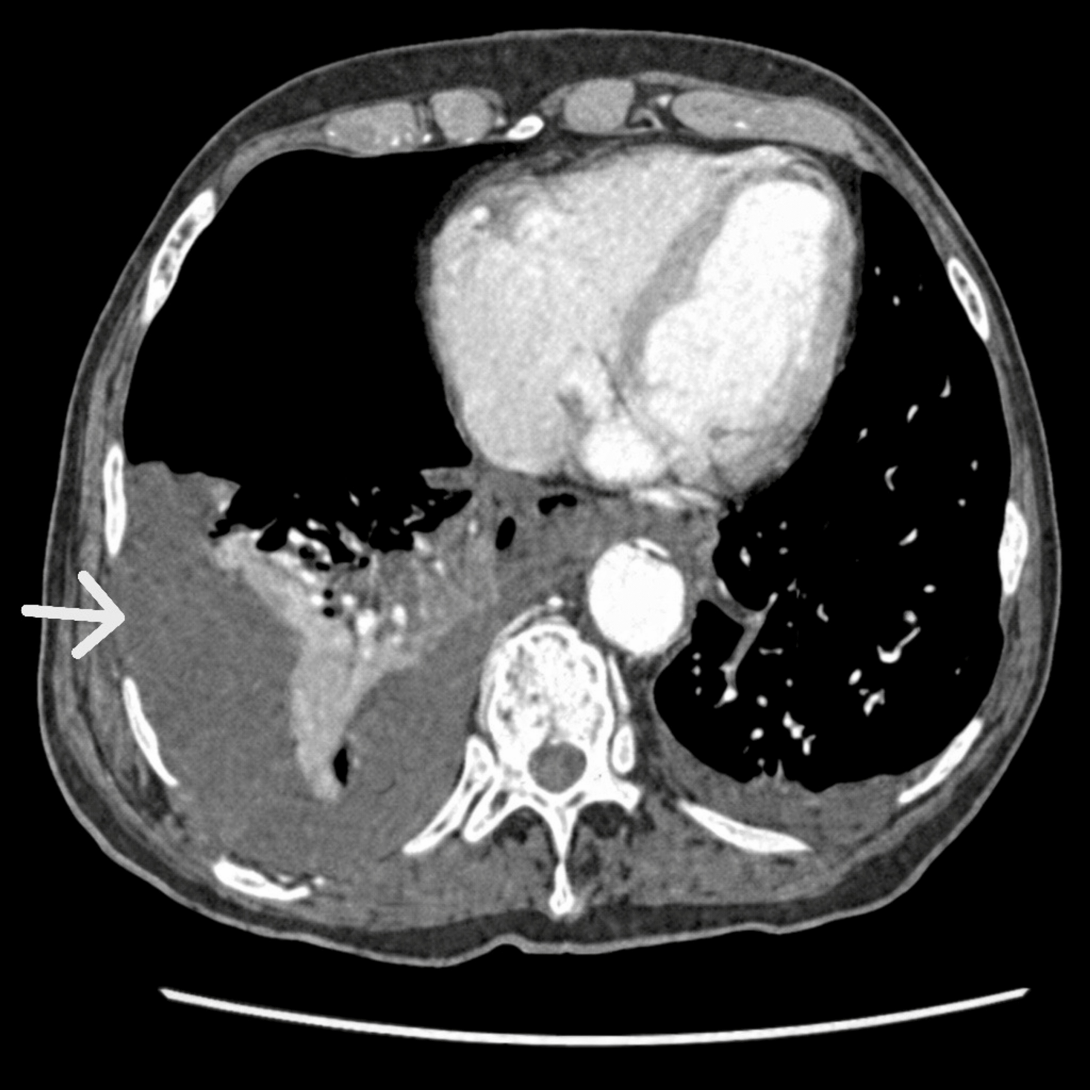
CT thorax (venous phase) shows a large right-sided hydropneumothorax with complete collapse of the right lung and mediastinal air posterior to the gastroesophageal junction (arrow), consistent with esophageal perforation.

During anesthesia, point-of-care ultrasound revealed a large right-sided pleural effusion. Subsequent CT thorax/abdomen showed a massive right-sided hydropneumothorax with complete collapse of the right lung, minimal left pleural effusion, posterior mediastinal air in relation to the gastroesophageal junction, and minimal ascites (Figures 2, 3). These findings were highly suspicious of esophageal perforation.

Despite aggressive stabilization and broad-spectrum antibiotics, the patient developed sepsis and rising inflammatory markers (CRP, 336 mg/L). Given her advanced malignancy, poor overall prognosis, and patient preference, invasive options such as esophageal stenting were not pursued. Following multidisciplinary discussion and in accordance with the patient’s wishes, further invasive interventions were withheld, and care was transitioned to a palliative approach.

## Discussion

Esophageal perforation is one of the most severe gastrointestinal emergencies, associated with high morbidity and mortality. Reported mortality ranges between 20% and 40%, largely depending on the time interval between perforation and initiation of treatment [2,4]. Early recognition and immediate management is therefore essential.

In the present case, the patient was admitted with massive hematemesis and profound hemodynamic instability, initially interpreted as variceal bleeding due to the dramatic clinical presentation. This illustrates an important diagnostic challenge: Boerhaave syndrome can mimic other causes of upper gastrointestinal bleeding, particularly in patients with risk factors or when vomiting precedes the event [3]. The presence of chest pain on admission was an important but nonspecific clue that should raise suspicion of esophageal perforation.

The classical Mackler’s triad of vomiting, chest pain, and subcutaneous emphysema is present in fewer than half of patients [5]. As in this case, atypical or overlapping symptoms frequently delay recognition. This emphasizes the need for a high index of suspicion, particularly in unstable patients with severe hematemesis and thoracic symptoms.

Although endoscopy plays a central role in the evaluation and acute management of upper gastrointestinal bleeding, its diagnostic utility in suspected esophageal perforation is limited. Visualization of a perforation may be obscured by active bleeding, clots, or edema, and the typical site of rupture, posterolateral in the distal esophagus, can be difficult to expose adequately. Furthermore, endoscopic insufflation may worsen existing perforation by increasing intraluminal pressure, potentially enlarging a tear or promoting mediastinal contamination. Several studies therefore caution that endoscopy can produce false-negative results in up to one third of patients [6]. In this case, significant active bleeding likely obscured the site of perforation, explaining the discrepancy between endoscopic and CT findings.

In contrast, CT imaging has become the diagnostic modality of choice for suspected esophageal perforation, with high sensitivity for detecting mediastinal air, pleural effusion, and associated thoracic complications [7]. In our patient, CT was decisive, revealing the massive hydropneumothorax, complete right lung collapse, and posterior mediastinal air that were not identifiable at endoscopy. Management strategies for esophageal perforation include primary surgical repair, endoscopic stenting, and conservative treatment with antibiotics and drainage [2,6,8-10]. Outcomes depend strongly on early recognition, extent of contamination, and patient comorbidity. In the present case, the patient’s advanced metastatic cancer, frailty, and rapid progression to sepsis made aggressive interventions unlikely to offer meaningful survival benefit. This aligns with literature emphasizing the importance of individualized treatment approaches in multimorbid patients [11]. Thus, the decision to pursue palliative management was appropriate and consistent with patient-centered care principles.

This case illustrates the diagnostic complexity of esophageal perforation in a patient initially suspected of variceal bleeding. While endoscopy is crucial for hemostasis in gastrointestinal bleeding, it may fail to identify perforation. Cross-sectional imaging, particularly CT, remains essential when clinical suspicion persists. Clinicians should maintain heightened suspicion when hematemesis is accompanied by chest pain or acute respiratory findings. In elderly, multimorbid patients, management decisions must balance potential benefit against overall prognosis and patient preference.

## Conclusions

Esophageal perforation should be considered in patients presenting with hematemesis and chest pain, even when variceal bleeding appears more likely. Endoscopy may fail to identify a perforation, whereas computed tomography can provide decisive diagnostic information. Management should be individualized and aligned with the patient’s overall prognosis and preferences, as invasive treatment may not be appropriate in multimorbid or palliative patients.

## References

[1] Derbes VJ, Mitchell RE (1955). Hermann Boerhaave’s Atrocis, nec descripti prius, morbi historia, the first translation of the classic case report of rupture of the esophagus, with annotations. Bull Med Libr Assoc.

[2] Brinster CJ, Singhal S, Lee L, Marshall MB, Kaiser LR, Kucharczuk JC (2004). Evolving options in the management of esophageal perforation. Ann Thorac Surg.

[3] Eroglu A, Can Kürkçüoǧlu I, Karaoǧlanoǧlu N, Tekinbaş C, Yımaz O, Başoǧlu M (2004). Esophageal perforation: the importance of early diagnosis and primary repair. Dis Esophagus.

[4] Biancari F, D'Andrea V, Paone R (2013). Current treatment and outcome of esophageal perforations in adults: systematic review and meta-analysis of 75 studies. World J Surg.

[5] Mackler SA (1952). Spontaneous rupture of the esophagus: an experimental and clinical study. Surg Gynecol Obstet.

[6] Chirica M, Champault A, Dray X, Sulpice L, Munoz-Bongrand N, Sarfati E, Cattan P (2010). Esophageal perforations. J Visc Surg.

[7] Young CA, Menias CO, Bhalla S, Prasad SR (2008). CT features of esophageal emergencies. Radiographics.

[8] Altorjay A, Kiss J, Vörös A, Bohák A (1997). Nonoperative management of esophageal perforations: is it justified?. Ann Surg.

[9] Biancari F, Saarnio J, Mennander A (2014). Outcome of patients with esophageal perforations: a multicenter study. World J Surg.

[10] Çarkıt S, İpekten F, Karaağaç M, Gök M, Akyuz M (2024). Esophageal perforation management: a single-center experience. Ulus Travma Acil Cerrahi Derg.

[11] Freeman RK, Ascioti AJ, Dake M, Mahidhara RS (2015). An assessment of the optimal time for removal of esophageal stents used in the treatment of an esophageal anastomotic leak or perforation. Ann Thorac Surg.

